# Stochastic Thermodynamics of a Linear Optical Cavity Driven on Resonance

**DOI:** 10.1021/acsphotonics.4c01401

**Published:** 2024-12-02

**Authors:** Vashist G. Ramesh, Joris Busink, René E. R. Moesbergen, Kevin J. H. Peters, Philip J. Ackermans, Said Rahimzadeh Kalaleh Rodriguez

**Affiliations:** Center for Nanophotonics, AMOLF, Science Park 104, XG Amsterdam 1098, the Netherlands

**Keywords:** nanophotonics, stochastic thermodynamics, optical cavity, fluctuations, fluctuation theorem

## Abstract

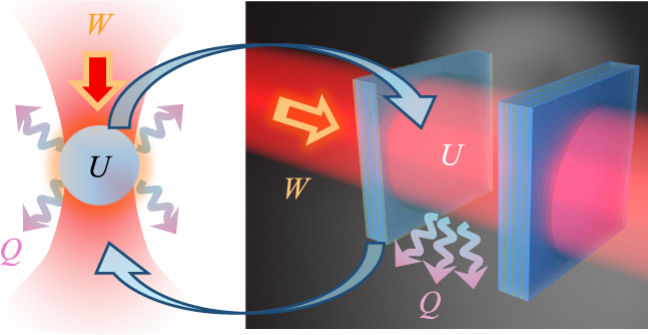

We present a complete framework of stochastic thermodynamics for a single-mode linear optical cavity driven on resonance. We first show that the steady-state intracavity field follows the equilibrium Boltzmann distribution. The effective temperature is given by the noise variance, and the equilibration rate is the dissipation rate. Next, we derive expressions for internal energy, work, heat, and free energy of light in a cavity and formulate the first and second laws of thermodynamics for this system. We then analyze fluctuations in work and heat and show that they obey universal statistical relations known as fluctuation theorems. Finite time corrections to the fluctuation theorems are also discussed. Additionally, we show that work fluctuations obey Crooks’ fluctuation theorem which is a paradigm for understanding emergent phenomena and estimating free energy differences. The significance of our results is twofold. On one hand, our work positions optical cavities as a unique platform for fundamental studies of stochastic thermodynamics. On the other hand, our work paves the way for improving the energy efficiency and information processing capabilities of laser-driven optical resonators using a thermodynamics based prescription.

## Introduction

Science usually precedes technology, but thermodynamics is an exception. Steam engines worked before thermodynamic laws were discovered. Actually, thermodynamics emerged from the desire to increase engine efficiencies. Eventually, the formulation and experimental validation of thermodynamic laws yielded more than better engines. Thermodynamics earned the timeless authority to determine which processes are possible, and to discard those ideas that do not abide by its principles. To date, technologies are conceived and optimized based on thermodynamics. This work is motivated by the conviction that many nanophotonic devices are now at a stage comparable to that of early steam engines. These devices can be made and characterized on astonishingly small scales thanks to nanotechnology, but a framework to increase their energy efficiency (with minimum sacrifice in speed and precision) in the inevitable presence of noise is lacking. The standard optics framework, based on deterministic Maxwell’s equations, cannot solve this issue because it neglects noise. However, a second chapter in the history of thermodynamics points at a solution.

**Figure 1:**
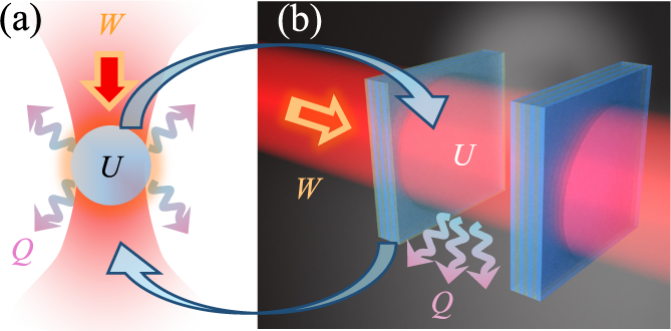
(a) A laser-trapped particle as widely studied in stochastic thermodynamics. *U* is the internal energy, *W* the work, and *Q* the heat dissipated to the environment. (b) A single-mode Fabry–Perót cavity, as studied in this manuscript. Notice how the roles of light and matter are reversed. In (a) the trapping potential is light, and the system of interest is made of matter. In contrast, in (b) the trapping potential is made of matter, while the system of interest is light.

Over the past 25 years, stochastic thermodynamics (ST) emerged as a comprehensive framework for describing small energy-harvesting and information-processing systems in contact with heat or chemical reservoirs.^1−4^ Consider, for example, a laser-trapped colloidal particle as shown in Figure 1a. This system works as a micron-scale heat engine,^5,6^ with laser and particle respectively replacing the piston and working gas. The first law of thermodynamics, δ*U* = *W* – *Q* relates the system’s change in internal energy δ*U* to the work *W* done on the system (usually negative for an engine) and the dissipated heat *Q*. ST is concerned with fluctuations in these and other thermodynamic quantities, which are prominent in small systems. By accounting for these fluctuations, ST places fundamental limits on energy and information processing capabilities of materials. ST advances the types of ideas needed to address current and emerging challenges in nanophotonics, many of which are related to stochastic effects. However, relations between optical and thermodynamic quantities need to be established.

Since photons typically do not reach thermal equilibrium, except under special conditions,^7^ thermodynamics is rarely used to describe states of light and their transformations. Recently, however, thermodynamics has been increasingly used to understand how material properties limit or enable optical functionalities.^8−14^ In addition, thermodynamic concepts have enabled the discovery and engineering of fascinating phenomena in multimode optical systems.^15−17^ Some aspects of stochastic quantum thermodynamics have been theoretically explored in optical resonators.^18^ However, a classical framework of stochastic thermodynamics has never been presented for a single-mode linear optical cavity. Filling this important knowledge gap is the goal of this manuscript.

Here we present a complete stochastic thermodynamic framework for a coherently and resonantly driven linear optical cavity. This manuscript is organized as follows. First we introduce the model for our system, and derive the scalar potentials confining light. In the second section, we show effective equilibrium behavior of light in a resonantly driven cavity. The steady-state intracavity field is shown to follow the equilibrium Boltzmann distribution, and an expression for the partition function is presented. In the third section, we formulate the first and second laws of thermodynamics for our system. Next, we analyze the averaged work and heat generated when modulating the laser amplitude. We elucidate how nonequilibrium behavior emerges when the modulation time is commensurate with the dissipation time. We then analyze work and heat fluctuations, and show that they obey universal statistical relations known as fluctuation theorems (FTs). We furthermore show that light in the cavity satisfies Crooks’ fluctuation theorem (CFT), enabling the estimation of free energy differences based on nonequilibrium work measurements. Finally, we summarize our results and discuss perspectives they offer.

## The Model

We consider a single-mode coherently driven linear optical resonator. We envision a laser-driven plano-concave Fabry-Perót cavity for concreteness, as illustrated in Figure 1b. However, our model equally describes any coherently driven resonator under three major assumptions. First, we assume that one mode is sufficiently well isolated, spectrally and spatially, from all other modes. Second, we assume that the laser intensity is sufficiently low for linear response to hold. Third, we assume that the physics under study is independent of the mode’s spatial structure. All three assumptions are frequently employed to successfully describe various optical resonators, such as open cavities,^19^ whispering-gallery-mode,^20^ photonic crystal,^21^ and plasmonic^22^ resonators. The third assumption, in particular, underlies the temporal coupled-mode theory that has enabled many important results in nanophotonics for decades.^23−26^ To date, temporal coupled-mode theory continues to inspire further scrutiny that, remarkably, continues to elevate its status as a useful theory.^27^

In a frame rotating at the laser frequency ω, the field α in the cavity obeys the following equation of motion:
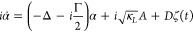
1 is the detuning between ω and the cavity resonance frequency ω_0_.  is the total loss rate, comprising the absorption rate γ_*a*_ and input–output rates  through the left and right mirrors. *A* is the laser amplitude, which we assume to be real.  is a stochastic force comprising a complex-valued Gaussian process .  and  have zero mean , autocorrelation , and cross-correlation . The constant *D* is the standard deviation of the stochastic force.

Our model accounts for two sources of noise in every coherently driven resonator. One of them is the noise of the incident laser. The other is the dissipative interaction of the cavity with its environment. According to the fluctuation–dissipation relation, that interaction results in fluctuations of the intracavity field. We can use a single pair of stochastic terms  to effectively account for both noise sources under the reasonable assumption that they are additive and Gaussian. Reference (28) presents one of many examples in the literature of an experimental system described by our model.

To analyze the deterministic force acting on α, we decompose eq 1 into real and imaginary parts. Setting , we get
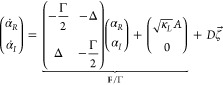
2eq 2 is a two-dimensional overdamped Langevin equation (OLE). The underbraced term contains the deterministic force **F**, divided by Γ to recover the normal form of the OLE. Notice that, unlike a standard OLE, eq 2 does not describe dynamics in position space. It describes instead Langevin dynamics in the phase space (α_*R*_, α_*I*_) of the optical resonator. Furthermore, α_*R*_ and α_*I*_ do not have units of length; they are unitless numbers. As a result, all thermodynamics quantities derived in this manuscript have units that deviate from convention. Nonetheless, a self-consistent effective thermodynamic framework can still be constructed as shown ahead.

**F** is fundamentally different when  or . When , **F** contains a conservative and a nonconservative part.^29^ A conservative force is one that can be derived from a scalar potential *U*, i.e., . A nonconservative force is equal to the curl of a vector potential: . This manuscript focuses entirely on the case , wherein the cavity is driven exactly on resonance and . Only in this case, an equilibrium steady-state can be expected.^29,30^

When , eq 2 decouples into a pair of independent one-dimensional OLEs:
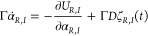
3The potential energies  are obtained by integrating the deterministic forces in eq 2:
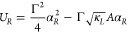
4a
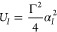
4bThe potentials are harmonic, as expected for a linear cavity. Their only difference is that the minimum of *U*_*R*_ is shifted from zero by the incident laser amplitude.

## Effective Equilibrium

**Figure 2:**
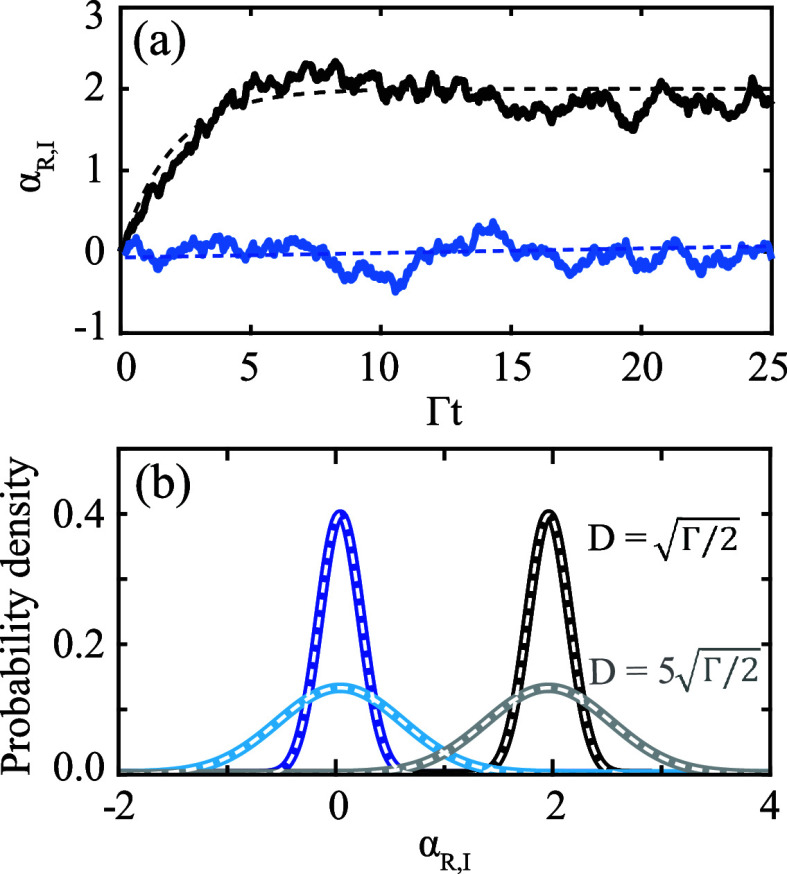
(a) Sample trajectories of the real and imaginary parts of the field, α_*R*_ and α_*I*_ in black and blue, respectively. Solid curves are numerical simulations of eq 3. Dashed curves are theoretical predictions from eq 5a and 5b. (b) Probability density functions (PDFs) of α_*R*_ and α_*I*_ in black and blue, respectively. Dark and light shades correspond to two different noise standard deviations *D*. White dashed curves are theoretical distributions using eqs 6 and 7a, 7b. Model parameters are , , , .

In Figure 2 we present numerical solutions to eq 3, obtained using the xSPDE MATLAB toolbox.^31^Figure 2a shows sample trajectories of α_*R*_ and α_*I*_ as black and blue curves, respectively. Based on 20000 of such trajectories, we constructed probability density functions (PDFs) of α_*R*_ and α_*I*_; these are shown as black and blue curves in Figure 2b, respectively. For both α_*R*_ and α_*I*_, we consider two different standard deviations of the noise *D*. In the following, we explain how Figure 2 displays effective thermodynamic equilibrium behavior. This behavior is present both at the level of the individual trajectories and of the PDFs.

Figure 2a shows α_*R*_ rising to a steady state and fluctuating thereafter. Meanwhile, α_*I*_ fluctuates around 0 (its steady state value) all the time. We can calculate the deterministic evolution of  by solving eq 3, with *D* = 0, analytically:
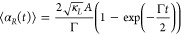
5a
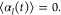
5b

The above solutions are plotted as dashed curves on top of the numerical results in Figure 2a. Notice that Γ^–1^ is the characteristic time in which α_*R*_ reaches its steady state. Since the steady-state distribution is the equilibrium Boltzmann distribution (shown next), Γ^–1^ is also the equilibration time of the fields.

The PDF of a gas in thermal equilibrium, confined in a scalar potential *U*, is the well-known equilibrium Boltzmann distribution . *Z* is the partition function, and  with *k*_*B*_ Boltzmann’s constant and *T* the temperature. Following Peters et al.,^29^ we can relate the noise variance *D*^2^ to the effective temperature of the light field via the fluctuation dissipation relation . Using this relation and the scalar potentials  in eq 4a and 4b, we arrive to the following expression for the Boltzmann distribution of the intracavity field:
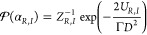
6

The partition functions can be obtained by imposing the normalization condition on eq 6, i.e., . Doing this, we get:
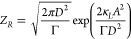
7a
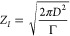
7b

Notice that both the equilibrium Boltzmann distribution and the partition functions are written in terms of the experimentally accessible standard deviation of the noise *D* and dissipation (cavity line width) Γ, instead of β which cannot be directly measured. In the next section we will use the expression for *Z*_*R*_ to formulate the second law of thermodynamics.

The white dashed lines in Figure 2b were calculated using eqs 6 and 7a, 7b. Their excellent agreement with the numerically calculated distributions demonstrates that light confined in a linear optical resonator displays effective thermal equilibrium behavior: the steady-state distribution is the equilibrium Boltzmann distribution. The effective temperature is related to the noise according to the aforementioned fluctuation–dissipation relation. For a detailed discussion about the meaning of the effective temperature *T*, we refer to ref^29^.

## First and Second Law of Thermodynamics

In this section we formulate the first and second law of thermodynamics for our resonantly driven linear optical cavity. Starting from eq 3, we apply the approach of Sekimoto^32^ to derive expressions for the internal energy, work, and heat. Since α_*R*_ and α_*I*_ are decoupled, we can treat them separately and obtain expressions for thermodynamic quantities in each case. In this vein, we first derive expressions for α_*R*_ (which will prove to be the more general case) and then for α_*I*_.

We begin by multiplying both sides of eq 3 with an infinitesimal field change :
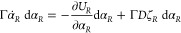
8Next, we use the expression for the total differential of , which according to the chain rule is . Using this expression for d*U*_*R*_, and the relation , we obtain from eq 8:
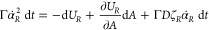
9We now substitute  and rearrange terms to get:
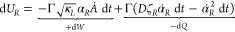
10Using the expression for *U*_*R*_ in eq 4a and integrating all terms in time (across an arbitrary trajectory from *t* = 0 to *t* = *s*) we get:
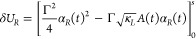
11a
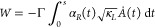
11b
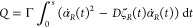
11cUsing the above expressions, we can now formulate the first law of thermodynamics for a resonantly driven stochastic linear optical cavity:
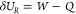
12

δ*U*_*R*_ is the net change in internal energy of the cavity over the trajectory. It comprises two terms. The first term is proportional to . For , as considered throughout this manuscript,  is also the number of intracavity photons. The second term is the force due to the laser  times the “displacement” α_*R*_ in phase space. This work-like contribution to the internal energy is present even in the absence of a protocol, i.e., when *A* is constant.

*W* is the work done by the laser field on the intracavity field. To recognize this, notice that the integrand in eq 11b contains the product of α_*R*_ and , which is the time derivative of the force due to the laser. Interpreting α_*R*_ as a displacement and integrating eq 11b results in a force times displacement, i.e., work. *W* > 0 means work is done on the intracavity field. The form of the work in eq 11b, introduced by Jarzynski,^33^ is in general different from the “classical work” as known in the statistical physics literature.^34,35^ The latter is defined as the time-integral of a force times a velocity. In particular, for *Ȧ* = 0 (constant laser amplitude) the so-called Jarzynski work is zero but the classical work is not. The two works are only equivalent for periodic driving , with τ the period.

The heat *Q* in eq 11c quantifies the transport of energy from the cavity to its environment. It contains two terms. The first term is the time-integrated dissipated power, given by the velocity squared as expected for a harmonic oscillator. The second term contains the product of the stochastic force  and the velocity , integrated over time. This is precisely the classical work done by the environment on the intracavity field. Thus, the net heat transfer is given by the difference between the dissipated energy to the environment and the work done by the environment on the system.

The units of *U*_*R*_, *W*, and *Q* all deviate from convention by a factor of Γ. Similarly, as evident in our fluctuation–dissipation relation , *k*_*B*_*T* has units Γ^2^. Thus, the extra factor of Γ also shows up in the thermal energy. The extra factor of Γ is due to the fact that while eq 1 has the form of the OLE, the dissipation Γ of our optical cavity is in the right-hand side of the equation. In contrast, the standard OLE reads , with γ the dissipation, **F** the deterministic force, and  the stochastic force. Besides the nonconventional units, our thermodynamic quantities also have a nonconventional interpretation because we describe Langevin dynamics in the phase space of the optical resonator rather than in position space. Our thermodynamic framework is thus effective in the following sense. The internal energy is not a function of position. It is a function of phase space distance relative to the fixed point. The work is not a force times a distance. It is rather a force times a distance in phase space. The same holds for the heat. It includes the work done in phase space by the bath, and the dissipated power in terms of a phase space velocity rather than a physical velocity. Despite the unconventional units and interpretation, the effective quantities we introduce are not mere mathematical constructs. They can be measured, they have a clear physical meaning related to phase space dynamics, and they are inter-related as prescribed by thermodynamics.

We now proceed to formulate the second law of thermodynamics for our system. The second law states
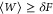
13with ⟨*W*⟩ the average work and δ*F* the free energy difference between initial and final states. The lower bound ⟨*W*⟩ = δ*F* is only attained by a reversible process. Notice that, unlike the first law, the second law does not hold at the level of individual trajectories. Actually, in the early days of stochastic thermodynamics, individual trajectories with *W* < δ*F* were occasionally called “transient violations of the second law”.^36−38^ Those were not really violations of the second law of course, which applies only on average.^2^

*W* for our resonantly driven stochastic linear optical cavity was already defined in eq 11b. Hence, to formulate the second law for our system we only need to define the free energy *F*. We can easily get this from the relation
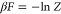
14with *Z* being *Z*_*R*_ or *Z*_*I*_ as defined in eqs 7a and 7b, respectively. Like the internal energy, *F* has units of *k*_*B*_*T*. It does not depend explicitly on time or α. It is therefore an intensive quantity that does not fluctuate. In the next section we show, through numerical simulations of our cavity system, that the second law is indeed always respected. However, in a finite-time trajectory there is a nonzero probability for . That probability is quantified by a fluctuation theorem.

Finally, we present expressions for thermodynamic quantities corresponding to α_*I*_. Notice from eqs 3 and 4a, 4b that the dynamics of α_*R*_ and α_*I*_ are similar. The only difference is the extra term containing the laser amplitude *A* in eq 4a. Thus, by setting *A* = 0 in eq 11a we get:
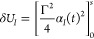
15a
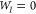
15b
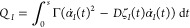
15cThe potential *U*_*I*_ is harmonic, as expected. In contrast to *U*_*R*_, the minimum of *U*_*I*_ is not shifted from zero by the laser amplitude. This simply means that the average internal energy (number of photons) of the degree of freedom that is not coupled to the driving laser, namely α_*I*_, is zero as expected. Similarly, the work done by the laser on α_*I*_ is zero because α_*I*_ is not coupled to α_*R*_ and the laser acts solely on α_*R*_. The only expression that remains qualitatively the same as for α_*R*_ is that of the heat. Energy can spontaneously flow in and out of the cavity because it is an open system, coupled to a (radiation) bath. As a result of these modified relations, the first law associated with α_*I*_ simply reads δ*U*_*I*_ = −*Q*_*I*_. Further, since no deterministic force does work on α_*I*_, the second law is irrelevant.

## Averaged Thermodynamic Quantities under Periodic Driving

In this section we discuss averaged thermodynamic quantities under time-harmonic driving. Protocols of this kind have been widely studied in stochastic thermodynamics.^4,39−42^ They have the benefit of generality—any protocol can be decomposed into sine and cosine modes via a Fourier transform.

**Figure 3:**
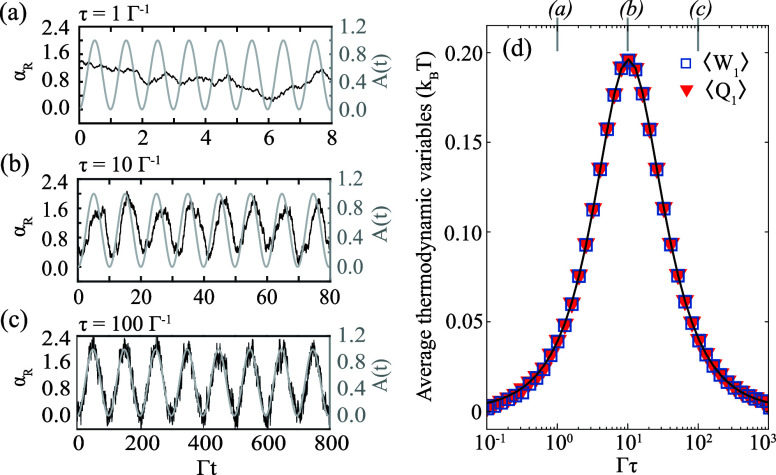
(a)–(c) Gray and black curves are the laser amplitude *A* and intracavity field α_*R*_, respectively. The modulation period τ is indicated in each panel. (d) Average work ⟨*W*_1_⟩ and heat ⟨*Q*_1_⟩ done in one modulation period of duration τ. The black curve is the theoretical prediction in eq 16. Model parameters: , , , .

Figure 3a–c show trajectories of α_*R*_ for one realization of the noise and three distinct τ. *A* and α_*R*_ are plotted as gray and black curves, respectively. Figure 3a was obtained for τ = Γ^–1^, which results in highly nonadiabatic dynamics. α_*R*_ cannot follow the driving laser because its instantaneous rate of change is too large compared to the equilibration time Γ^–1^. Next, Figure 3b was obtained for τ = 10 Γ^–1^. Here the trajectory of α_*R*_ resembles the driving protocol, but there is a delay which results in hysteresis. This hysteresis is due to the fact that α_*R*_ does not fully equilibrate at any point in time during the protocol. While the period exceeds the equilibration time, the amplitude of the modulation is so large that the system is constantly driven out of equilibrium. Finally, Figure 3c was obtained for τ = 100Γ^–1^. For this slow protocol, α_*R*_ approximately follows the laser and departures from equilibrium are minimal. Overall, Figure 3a–c illustrate how nonequilibrium behavior emerges when the intracavity field changes within a time that is commensurate with, or shorter than, Γ^–1^.

In Figure 3d we analyze the average work and heat (as defined in eq 11a–c) produced in one period of the modulation in *A*. Averages are done over 2000 modulation periods, and we plot the results as a function of τ. Notice that the average work and heat are always equal to each other. This is a consequence of the first law combined with a net zero change in average internal energy. The average internal energy does not change because initial and final states in our periodic protocol are the same. Further, notice in Figure 3d that , as expected from the second law. Moreover, the lower bound  is attained in the adiabatic limit , wherein the system remains in equilibrium and the dynamics are reversible.

On top of the numerical simulations in Figure 3d, we plot the theoretical prediction for the average work and heat as a black curve. This was obtained by setting  and *D* = 0 in eqs 3 and 11a–c, which results in the expression
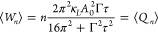
16The subscript *n* is the number of modulation periods integrated over, which is equal to one for the results in Figure 3d. The theoretical prediction is in excellent agreement with the simulations.

Figure 3d shows that ⟨*W*_1_⟩ and ⟨*Q*_1_⟩ depend nonmonotonically on τ. Both quantities follow a Lorentzian function, in agreement with eq 16. We identify three regimes depending on τ. In the adiabatic limit , the dynamics are reversible, the system remains in equilibrium, and . In the nonadiabatic regime , the dynamics are irreversible, the system in driven far from equilibrium, and ⟨*W*_1_⟩ is maximized. In the limit , the dynamics are still nonequilibrium but . The work vanishes because the driving protocol *A*(*t*) is so fast that α cannot respond to *A*(*t*).

## Fluctuation Theorems

### Symmetry Functions

While the second law demands ⟨*W*⟩ ≥ 0, individual trajectories can yield *W* < 0. At the heart of this possibility is the time-reversibility of microscopic dynamics. A solution to the OLE yielding +*W* has a time-reversed counterpart yielding −*W*. However, the probabilities of observing + *W* and −*W* are not equal. The ratio of these probabilities is determined by a fluctuation theorem (FT). Simply put, FTs are the extension of the second law to stochastic systems. They transform the inequality in the second law into an equality for the probability ratios of realizing positive and negative work, or positive and negative entropy production in general.^1^

FTs can generally be expressed in the form of a symmetry function,^4^
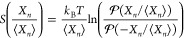
17*X*_*n*_ can represent work *W*_*n*_ or heat *Q*_*n*_, and ⟨*X*_*n*_⟩ its average.  and  are the probability of positive and negative *X_n_*/⟨*X*_*n*_⟩, respectively. Thus, the symmetry function quantifies the asymmetry between the negative and positive regions of the PDF of *X*_*n*_. Here, inspired by the works of Ciliberto et al.^42^ and Cohen et al.^43^ for mechanical and electrical oscillators, we calculate the symmetry functions of the work *W*_*n*_ and heat *Q*_*n*_ for our linear optical cavity driven on-resonance by a time-periodic laser amplitude.

We focus on a particular class of FTs that describes nonequilibrium fluctuations around a steady state, the so-called Steady-State Fluctuation Theorem (SSFT).^44−46^ In terms of the symmetry functions, the SSFT predicts that  in the extensive limit . Essentially, the SSFT states that negative fluctuations of *X*_*n*_/⟨*X*_*n*_⟩ are exponentially suppressed as *X*_*n*_/⟨*Xn*⟩ grows. At finite time the following linear relationship is assumed:
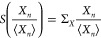
18The slope  measures finite-time deviations from the SSFT. If , the SSFT holds exactly.^41^

**Figure 4:**
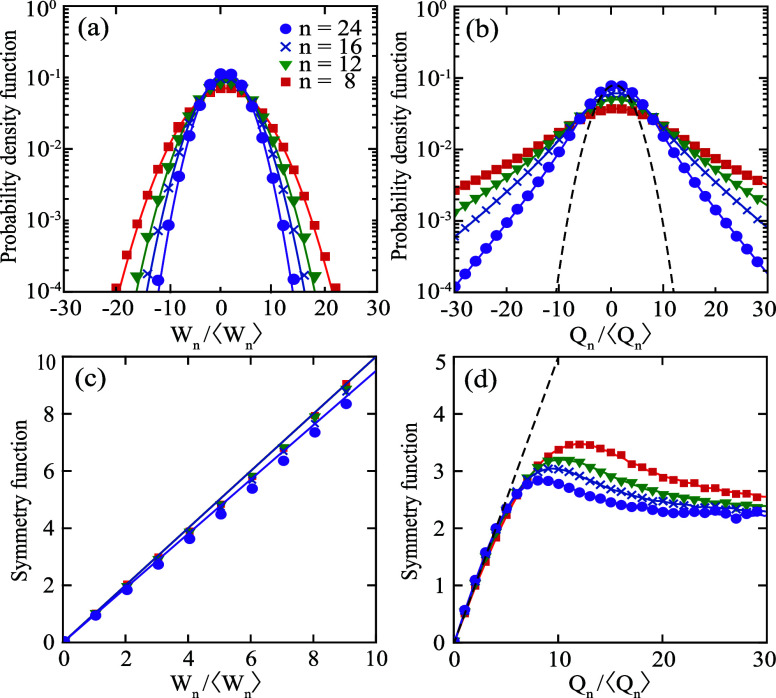
Probability distribution functions of (a) the rescaled work *W*_*n*_/⟨*W*_*n*_⟩ and (b) the rescaled heat *Q*_*n*_/⟨*Q*_*n*_⟩, for varying number of cycles *n* integrated over. Symbols represent numerical data. Solid curves are Gaussian fits in (a) and linear interpolation in (b). The dashed black curve in (b) is a Gaussian distribution fitted to the numerical data for *n* = 24 in the neighborhood of *Q_n_*/⟨*Q*_*n*_⟩ = 1. Below their respective PDFs, the symmetry functions of *W*_*n*_/⟨*W*_n_⟩ and *Q*_*n*_/⟨*Q*_*n*_⟩ (from eq 17) are shown in (c) and (d). Model parameters are the same as in Figure 3, except for τ = 100Γ^–1^ and .

We now elucidate the FT and symmetry functions through numerically calculated PDFs of work and heat. These are presented in Figure 4a,b, respectively. We obtained these PDFs from 10000 trajectories of α_*R*_, each comprising 24 cycles of a time-harmonic protocol in *A*(*t*) with period τ = 100Γ^–1^. We include 4 different PDFs in Figure 4a,b, corresponding to a different number *n* of cycles over which the work or heat are calculated. PDFs are centered at 1 because both heat and work are divided by their average values.

Work distributions are Gaussian. Indeed, the solid curves Figure 4a are Gaussian distributions perfectly fitting the numerical data. Heat distributions, in contrast, are approximately Gaussian for small fluctuations only. To evidence this, in Figure 4b we fitted a Gaussian distribution to the numerical data for *n* = 24 in the neighborhood of *Q_n_*/⟨*Q*_*n*_⟩ = 1. For , all heat PDFs are non-Gaussian. Instead, they depend linearly on *Q_n_*/⟨*Q*_*n*_⟩ in the log–linear scale, meaning the distributions have exponential tails.

Notice that both work and heat distributions become narrower as *n*, and therefore the integration time, increases. Accordingly, the probability of observing large negative fluctuations (the so-called “transient violations of the second law”) decreases. Further, notice that large fluctuations are more likely in the heat than in the work.  is wider than  for all *n*. We can understand this different behavior in view of the expression for the heat, eq 11c, which contains two quantities. The quantity  is the work done by the bath on the system. It is linear, and dominates the heat when fluctuations in α_*R*_ are small. As a result, small heat fluctuations are work-like and Gaussian distributed. The other quantity, , is the power dissipated within a harmonic potential, which is quadratic in α_*R*_. This nonlinear term dominates the heat when fluctuations in α_*R*_ are large. These large heat fluctuations are exponential distributed, just like the potential energy fluctuations of an harmonic oscillator.^47^ We thus see that the difference in shape of work and heat PDFs is due to the complex interplay between power dissipation and energy transfer to the cavity that is present in the heat, but not in the work. From a mathematical perspective, large fluctuations are more likely to occur in the heat than in the work simply because the exponential falls off more slowly than the Gaussian. We stress that the different statistical properties of work and heat are not particular to our optical system. They are simply the result of having a harmonic potential.

Using eq 17, with , we can now calculate the symmetry functions of work and heat. These are presented in Figure 4c and 4d, respectively. The slope  of the work symmetry function is equal to 1 for any number of cycles integrated over. However, the heat symmetry function is very different. First, the symmetry function of heat is only linear in the region of small *Q*_*n*_/⟨*Q*_*n*_⟩. Therein, the slope  of the symmetry function is 0.5. Second, for large fluctuations the symmetry function is nonlinear and converges to ≈2 for very large fluctuations. This means that large negative fluctuations in *Q*_*n*_ are still relevant compared to large positive fluctuations.

Overall, Figure 4c,d show that *W*_*n*_ follows the standard SSFT exactly, while *Q*_*n*_ does not. Instead, *Q*_*n*_ follows an extended form of the SSFT developed by van Zon and Cohen.^48^ Under nonadiabatic driving, deviations from the SSFT (and its extension) arise due to finite time effects, as shown next.

### Finite Time Corrections

**Figure 5:**
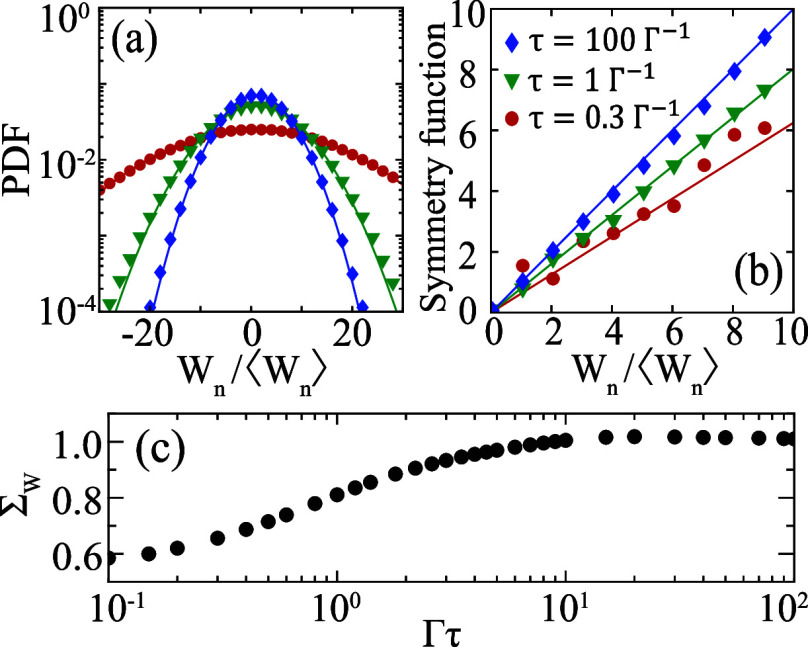
(a) Probability distribution function of the rescaled work *W_n_*/⟨*W*_*n*_⟩ under time-harmonic driving. The period τ is indicated in the legend of (b). Symbols are numerical data, solid curves are Gaussian fits. (b) Work symmetry functions using the same color scheme. Solid lines are symmetry functions obtained from the Gaussian fits to the PDFs. (c) The slope  of the symmetry function as a function of Γτ. Parameters are the same as in Figure 4, with *n* = 8.

We now analyze finite time corrections to the SSFT when the period τ of the driving protocol becomes comparable to the equilibration time Γ^–1^. We consider a fixed number of cycles *n* = 8. In Figure 5a we compare PDFs of the rescaled work *W*_*n*_/⟨*W*_*n*_⟩ for three distinct τ, indicated in the legend of Figure 5b. The PDFs are Gaussian for all τ, as expected. They broaden as τ decreases. Large fluctuations become increasingly relevant in fast protocols.

Figure 5b shows the work symmetry function for three different τ. Notice how the slope  of the symmetry function decreases as τ decreases. In Figure 5c we plot  across a wide range of τ.  is less than 1 for small τ, but converges to 1 in the adiabatic limit . The change in  quantifies the finite time corrections to the SSFT for nonadiabatic driving.

**Figure 6:**
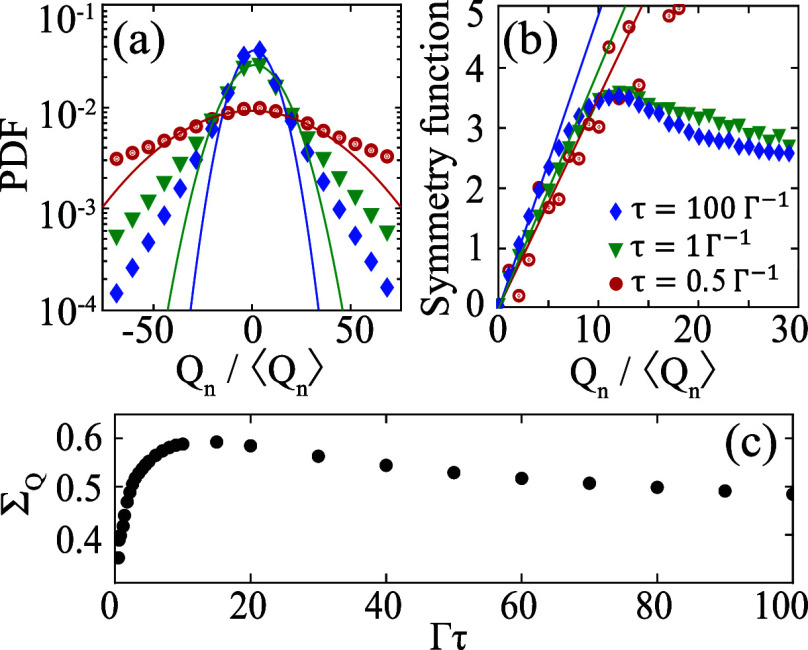
Same as in Figure 5 but for the rescaled heat *Q*_*n*_/⟨*Q_n_*⟩ instead of the work. Parameters are the same as in Figure 5.

Figure 6 presents a similar analysis to the one in Figure 5, but now for heat instead of work. Figure 6a shows the PDFs. They are approximately Gaussian for small ±*Q*_*n*_, but exponential at the tails. As τ decreases the PDFs broaden and the Gaussian region widens. This significantly changes the symmetry function, as Figure 6b shows. In particular, the symmetry function becomes linear over a wider range of *Q*_n_/⟨*Q*_*n*_⟩ for small τ. In Figure 6c we plot the slope  of the heat symmetry function. We observe a nonmonotonic dependence on τ.  converges to 0.5 in the adiabatic limit, consistent with the results in Figure 4.

### Crooks’ Fluctuation Theorem

We previously studied FTs for a protocol starting and ending in the same equilibrium steady state. Now we demonstrate a FT for the work done during the forward and backward parts of such a protocol; by forward and backward parts we mean the half-cycles whereby the laser amplitude increases and decreases, respectively. In particular, we demonstrate Crooks’ fluctuation theorem (CFT)^49^ for our coherently driven linear optical resonator. The CFT is a paradigm for understanding emergent phenomena.^4,50,51^ It enables estimating free energy differences by measuring forward and backward work PDFs. Crucially, the CFT holds regardless of the speed of the process, and hence on how far from equilibrium the system is driven. However, the CFT assumes that the system starts and ends in equilibrium.

Consider a driving protocol that takes a system from initial to final state and back symmetrically. Then, the CFT states that
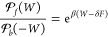
19*W* is the work.  is the probability of +*W* being generated in the forward half-cycle and  is the probability of −*W* being generated in the backward half-cycle. δ*F* is the free energy difference between initial and final states.

Essentially, eq 19 quantifies the reversibility of a transition between two equilibrium steady states. It does so in terms of the asymmetry of work distributions in the forward and backward directions. The crossing point of the two distributions, i.e., the value of *W* for which =, is exactly δ*F*. This possibility, namely to estimate equilibrium free energies by performing nonequilibrium measurements, is possibly the main reason for which the CFT became a pillar of stochastic thermodynamics.

The CFT has been used to measure free energy differences in single molecules^34,50,52,53^ and mechanical systems.^35^ Here we use it in the context of our coherently driven linear optical resonator. To this end, we performed numerical simulations of eq 3 with a time-harmonic protocol in the laser amplitude. *A* increases from 0 to  in the forward part of the protocol, and decreases from  to 0 in the backward part. The period is τ = 200Γ^–1^. For each half-cycle we calculated the work done using eq 11b. Finally, we obtained the distributions  and  from an ensemble of 10000 independent cycles.

**Figure 7:**
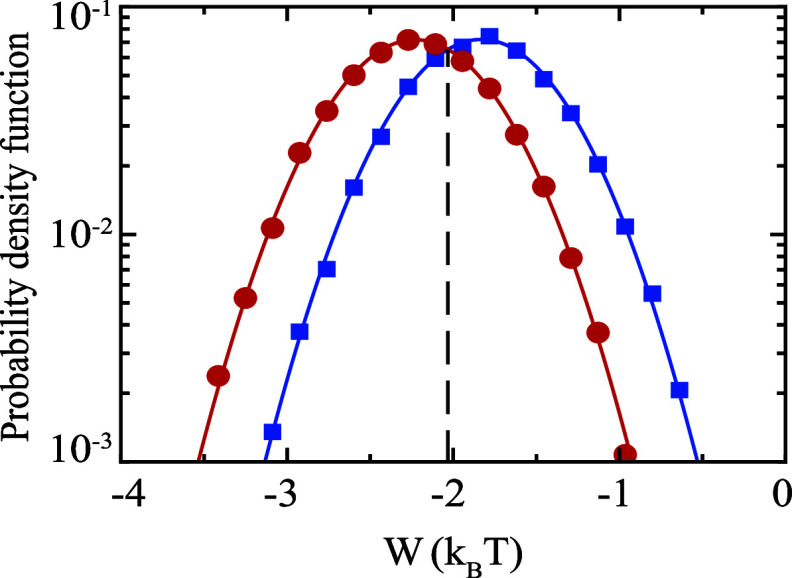
Blue squares and red dots indicate probability distributions of the work done during the forward and backward half-cycles along a time-harmonic protocol in the laser amplitude, respectively. Solid curves are Gaussian fits. The vertical dashed line indicates the intersection point of the PDFs, which marks the free energy difference between the initial and final states according to eq 19. Model parameters are same as in Figure 3 except: τ = 200Γ^–1^, , .

Figure 7 shows the forward and backward work distribution as red and blue dots, respectively. The two distributions intersect at *W* = −2.041 *k*_*B*_*T*, as indicated by the dashed line in Figure 7. According to the CFT, this is exactly the free energy difference between the steady states at the start and end of our protocol in *A*. We can verify this result by calculating free energies of those states using eq 14. Indeed, inserting the parameters reported in Figure 7 into eq 14, we exactly calculate the free energy difference between initial and final states of our protocol to be δ*F* = −2.041 *k*_*B*_*T*. We highlight that while the results presented in this manuscript were obtained for a large driving period, we verified that the results are independent of the period. However, if a small (compared to Γ^–1^) driving period is used, the system needs to be allowed to equilibrate by halting the protocol for some time at the start and end of each half-cycle.

## Conclusion and Perspectives

In summary, we presented a complete framework of stochastic thermodynamics for a single-mode linear optical cavity driven on resonance. We showed that light in such a cavity displays effective equilibrium behavior. We formulated the first and second laws of thermodynamics for the cavity in terms of optical control parameters and observables. We analyzed the work and heat generated when the cavity is driven by a periodically modulated laser amplitude. The averaged work and heat produced per cycle is maximized when the driving period is commensurate with the equilibration time of the system and the dynamics are strongly irreversible. Further, we discussed fluctuation theorems for work and heat, including their finite time corrections. Finally, we showed how measurements of forward and backward work can enable the estimation of free energy differences between optical states via Crooks’ fluctuation theorem.

Our work opens a new research avenue at the crossroad between stochastic thermodynamics and nanophotonics. To date, nanophotonics has primarily provided tools for probing stochastic thermodynamics of material systems in new regimes. A prime example of this is in the field of levitodynamics, where sophisticated nanophotonic methods are used to trap particles in new settings,^54^ or to introduce entirely new types of particles in the trap.^55^ Here, in contrast, we completely reversed the role of light and matter: our trap is made of matter, while light is the stochastic thermodynamic system. This difference opens intriguing opportunities for new fundamental physics studies and technological applications.

Fundamentally, optical cavities can facilitate probing fluctuation theorems for systems relaxing to a nonequilibrium steady state (NESS).^2,30^ The strength of nonequilibrium behavior can be controlled via the laser-cavity detuning. As the detuning deviates from zero, a nonconservative force in the optical phase space leads to dynamics resembling two-dimensional Brownian motion in a stirred fluid.^28^ The nonconservative force makes it impossible to define a path-independent work, and the heat contains an additional contribution (the so-called housekeeping heat) associated with maintaining the NESS.^56^ Consequently, we expect several modifications to the results in this manuscript. In particular, the CFT will not hold. We expect instead new fluctuation theorems for generalized heat-like and work-like thermodynamic quantities.^30^ The derivation of such fluctuation theorems could be achieved via the path-integral formalism for general Langevin processes,^30^ or via the master equation approach.^57^ Either way, this is a major theoretical effort that, if successful, could shed light on fundamental bounds that thermodynamics places on nanophotonic devices driven at any frequency.

A strength of optical cavities in the context of probing fluctuation theorems is the extremely wide dynamic range they offer, which is ideal for characterizing rare events. For example, a single-mode cavity with Kerr nonlinearity can take longer than the age of the universe to relax to its steady state.^58,59^ Meanwhile, relevant dynamics unfold within the dissipation time, typically on the order of a picosecond. Thus, within a single second one can in principle (pending practical limitations) attain statistics of dynamics spanning 12 orders of magnitude in time. No other system can give access to such a wide dynamic range and with such ease. The access to rare events that this extraordinary dynamic range offers can be particularly useful for characterizing non-Markovian dynamics, which is theoretically challenging. Experimentally, in contrast, non-Markovian dynamics can be easily realized by introducing thermo-optical nonlinear media inside a cavity.^60,61^

Finally, we foresee exciting technological opportunities in the extension of stochastic thermodynamic concepts to optical cavities. For instance, optimal protocols could be designed to drive an optical system from one state to another with minimum dissipation.^62^ Alternatively, time-information uncertainty relations^63^ could be used to establish speed limits for transitions between optical states, and thus for optimizing optical devices using such transitions. For these and other directions leveraging stochastic thermodynamics to bound optical functionality, it may be necessary to go beyond the present framework and account for spatial effects. This could be achieved along the lines of the recently developed spatiotemporal coupled mode theory,^64^ which upgrades the standard temporal coupled-mode theory to account for spatial effects. In summary, we expect many fascinating opportunities to emerge from the recognition that resonant optical systems can be described, and eventually optimized, like light engines.
